# A rare cause of jejunal perforation: Monomorphic epitheliotropic intestinal T‐cell lymphoma

**DOI:** 10.1002/jgh3.12844

**Published:** 2022-11-16

**Authors:** Masahiro Yanagi, Takuya Komura, Takashi Kagaya

**Affiliations:** ^1^ Department of Gastroenterology National Hospital Organization Kanazawa Medical Center Kanazawa Japan

**Keywords:** jejunum, monomorphic epitheliotropic intestinal T‐cell lymphoma, perforation

## Abstract

Monomorphic epitheliotropic intestinal T‐cell lymphoma (MEITL) is a very rare intestinal T‐cell lymphoma which is observed most frequently in the jejunum. MEITL is prone to cause intestinal perforation and the prognosis is very poor when it occurs. Here we report a fatal case of MEITL causing jejunal perforation at the time of diagnosis in a 79‐year‐old man. The patient underwent emergency surgery for jejunal perforation caused by MEITL but died 3 months after the initial visit due to prolonged peritonitis. It is desirable to establish a method to predict cases with intestinal perforation, and systematize the treatment strategies to avoid perforation.

## Graphical abstract

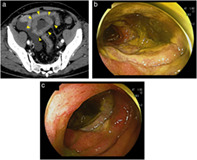

## Introduction

Intestinal perforation and peritonitis are known complications of intestinal tumors that can occur either at diagnosis or during treatment. They can be life‐threatening and worsen the prognosis due to sepsis, multiple‐organ failure, prolonged hospitalization, or delays in the initiation of chemotherapy.^1^

Among the primary malignant tumors of the small intestine, intestinal perforation frequency is reported to be 11.5%–14% in malignant lymphoma, 5.9% in leiomyosarcoma, and 2% in carcinoma.^2^ Malignant lymphomas are at high risk of intestinal perforation. Among intestinal malignant lymphomas, a higher incidence of intestinal perforation has been reported in T‐cell lymphomas compared with B‐cell lymphomas.^3^ Perforation can occur at the initial presentation of malignant lymphoma or after the initiation of chemotherapy.^1^

Here we report a fatal case of monomorphic epitheliotropic intestinal T‐cell lymphoma (MEITL) causing jejunal perforation at the time of diagnosis in a 79‐year‐old man.

## Case report

**Figure 1:**
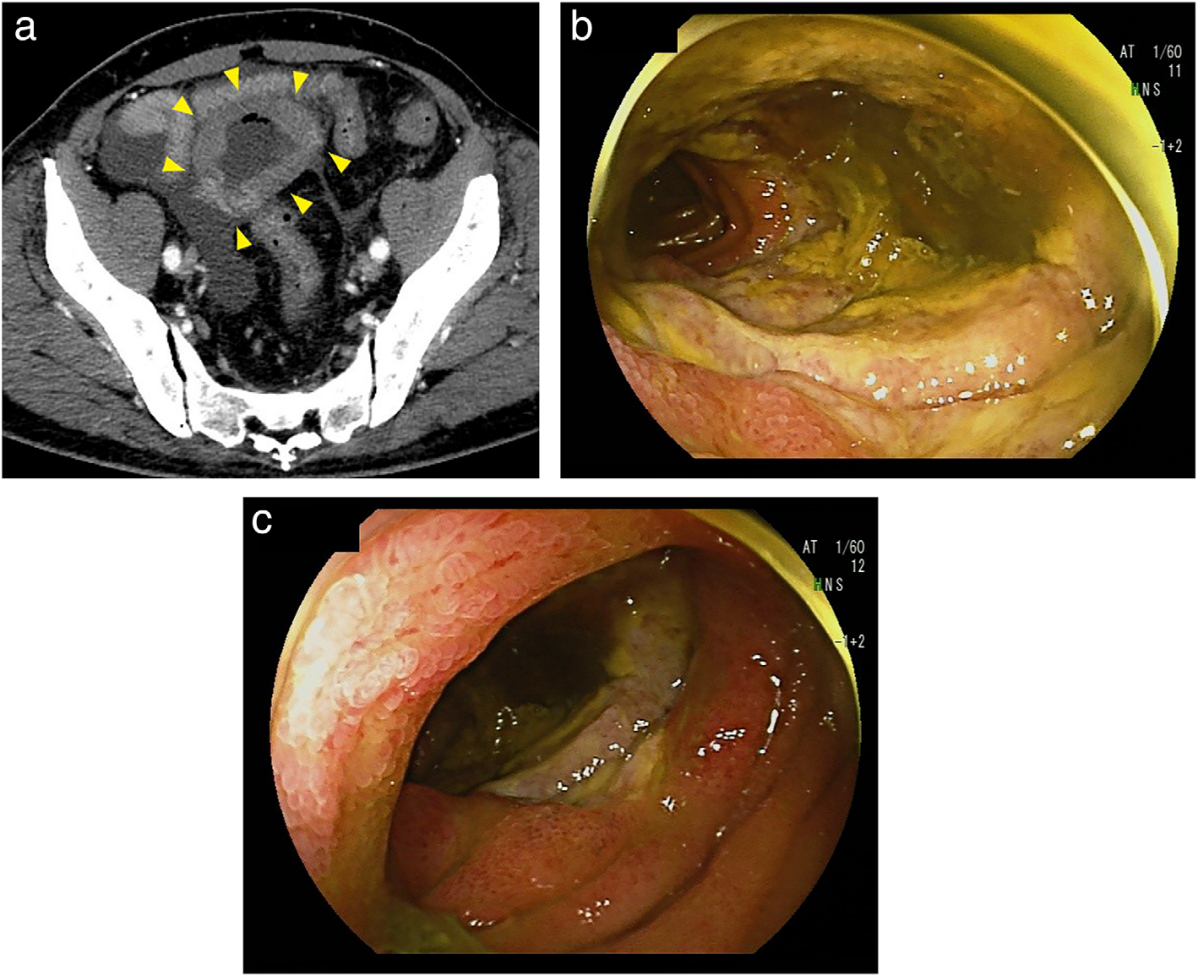
(a) Contrast‐enhanced abdominal CT showing circumferential wall thickening and luminal dilatation in the jejunum (arrowheads). (b) Endoscopic view of the jejunum showing a deep, nearly circumferential ulcer. The ulcer base was covered with a thick slough and (c) the ulcer was surrounded by a granular membrane with swollen villi.

**Figure 2:**
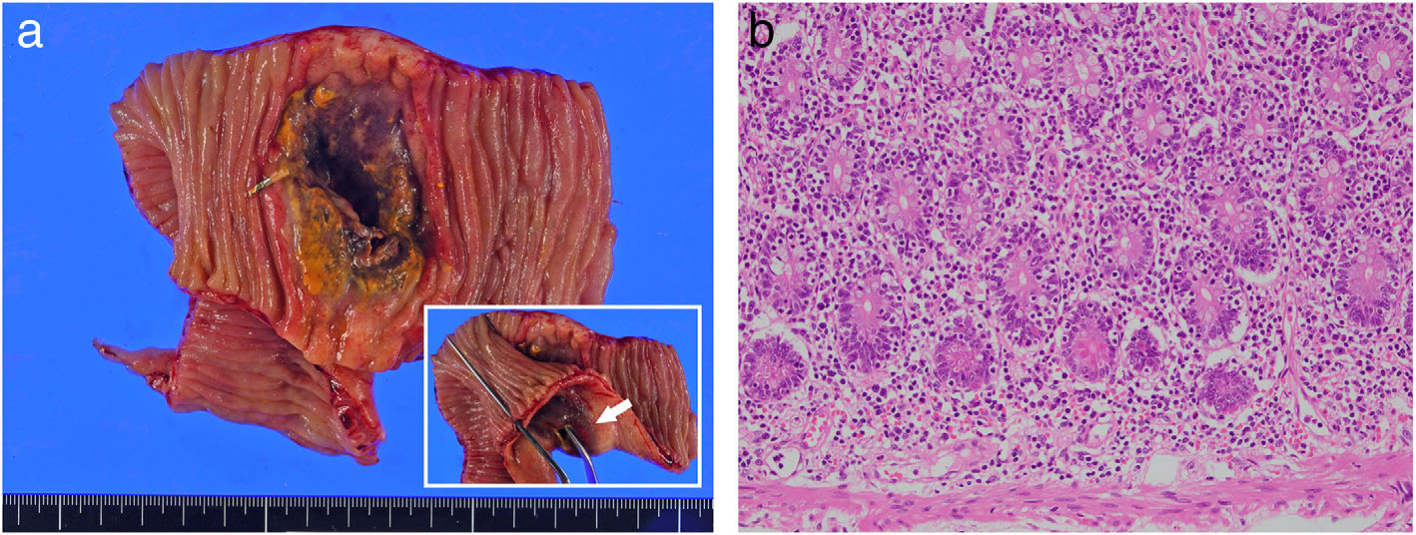
(a) Resected specimen showing a nearly circumferential ulcerative lesion with a fistula at the deepest point (arrow). (b) Histopathology with diffuse transmural infiltration of small‐to‐medium‐sized tumor cells (H&E, ×200). The cells were positive for CD3, CD8, and CD56 (not shown).

A 79‐year‐old male patient presented with a history of 5 kg weight loss in 1 year. Contrast‐enhanced abdominal computed tomography (CT) showed a 5‐cm‐long segment of the jejunum with circumferential wall thickening and luminal dilatation (Fig. 1a). There were no extraintestinal lesions on CT. Laboratory tests indicated anemia with a hemoglobin level of 10.7 g/dL, hypoalbuminemia with an albumin level of 2.1 g/dL, and elevation of soluble interleukin‐2 receptor level (1780 U/mL; normal, <474 U/mL). Trans‐anal double‐balloon enteroscopy revealed a deep, nearly circumferential ulcer in the jejunum. The ulcer base was covered with a thick slough (Fig. 1b), and the ulcer was surrounded by a granular membrane with swollen villi (Fig. 1c). Biopsies were performed at the ulcer margin for pathological evaluation. The patient was scheduled for positron emission tomography (PET/CT) but was unexpectedly admitted to the emergency room 2 weeks later due to sudden‐onset severe abdominal pain. Abdominal CT showed massive ascites and free air. Emergency surgery was performed due to suspicion of gastrointestinal perforation. Surgical findings showed a perforated area in the jejunum, on the anorectal side, 150 cm distal to the Treitz ligament. Apart from the main lesion, a small lesion without perforation was found on the anorectal side, 50 cm distal to the Treitz ligament. Segmental resection of the perforated jejunum was performed. The surgical specimen showed a nearly circumferential infiltrative ulcerative mass (72 × 34 mm) with a 21 mm fistula at the deepest point (Fig. 2a). Pathologic findings revealed diffuse transmural infiltration of small‐to‐medium‐sized CD3^+^, CD8^+^, and CD56^+^ tumor cells (Fig. 2b). Tumor cells were positive for Granzyme B, a cytotoxic factor, and the Ki‐67 index was high at 90%. EBV‐encoded small RNA (EBER) was negative on in situ hybridization. Diffuse tumor cells infiltration was also seen in the adjacent mucosa. Transmural necrosis was observed at the perforation site. In addition, the background mucosa distant from the tumor showed no evidence of villous atrophy or increased intraepithelial lymphocytes (IELs), characteristic of celiac disease. The final diagnosis was MEITL. After surgery, however, the patient continued to suffer from peritonitis as a result of gastrointestinal perforation, and his general condition gradually worsened. Consequently, the patient was deemed ineligible for chemotherapy and was transferred to home care. He died 3 months after the initial visit.

## Discussion

Intestinal T‐cell lymphoma is a very rare tumor, accounting for 0.25% of all malignant lymphomas and less than 5% of primary malignant lymphomas of the digestive tract.^4^

In the revised fourth edition of the WHO classification published in 2017, conventional enteropathy‐associated T‐cell lymphoma (EATL) type I associated with celiac disease is called EATL, and conventional EATL type II not associated with celiac disease is called MEITL.^4^ The latter is a rare, rapidly progressive, primary intestinal T‐cell lymphoma that arises from intestinal intraepithelial T lymphocytes and is most common in the proximal small bowel, especially the jejunum.^5^, ^6^ It is found predominantly in Asian and Hispanic populations, with a median age of onset of about 60 years.^5^, ^6^ Its typical symptoms include abdominal pain, diarrhea, and weight loss, and approximately 50% cases are diagnosed with acute abdomen due to intestinal perforation or obstruction.^5^, ^7^ The endoscopic findings show single or multiple ulcers, edematous mucosa, mucosal thickening, and luminal stenosis.^8^ MEITL ulcers are characterized by a surrounding edematous or granular mucosa due to infiltration of tumor cells into the peri‐ulcerative mucosa.^8^ Pathologically, MEITL is characterized by diffuse transmural proliferation of CD8^+^ and CD56^+^ tumor cells. It has also been reported that MEITL cells express high levels of cytotoxic factors (e.g., Granzyme B, T1A1, and perforin).^9^

Intestinal T‐cell lymphoma, especially the ulcerative type, causes destructive transmural infiltration without proliferation of connective tissue, making the tumor itself extremely fragile and prone to cause intestinal perforation.^10^ In addition, intestinal T‐cell lymphoma shows vaso‐centric infiltration of tumor cells, resulting in tissue degradation and necrosis, which is another predisposing factor to intestinal perforation.^11^ The T‐cell markers CD8^+^ and CD56^+^ have been reported as indicators of intestinal perforation in intestinal T‐cell lymphomas: intestinal perforation is more common in CD8^+^ or CD56^+^ cases than negative cases.^12^, ^13^

According to a report of 63 cases of intestinal T‐cell lymphoma with small intestinal perforation by Kawai et al., the median age was 63 (5–90) years, the male‐to‐female ratio was 38:25, and the most common site of perforation was the jejunum, accounting for 69.4% (43 of 62 cases). Exactly 71.7% (38 of 53 patients) had multiple lesions and 25% (10 of 40 patients) had multiple perforations. The positivity rates of CD8^+^ and CD56^+^ were 80% and 69.7%, respectively.^12^

The present case had multiple lesions and a single perforation. Histopathologically, infiltration of CD8^+^ and CD56^+^ tumor cells was documented with high cell proliferation (Ki‐67 index 90%) and secretion of a cytotoxic factor (Granzyme B). These formed a huge ulcerative mass with transmural necrosis, suggesting strong tissue destruction and blood flow disturbance; the risk of intestinal perforation was considered very high.

The prognosis of intestinal T‐cell lymphoma has been reported to be very poor, with a 1‐year survival rate of 38.7% and a 5‐year survival rate of 19.7%. Median survival for cases with small intestinal perforation is even poorer, at 4 months (3 days–75 months),^12^ suggesting that in addition to chemotherapy resistance of the tumor itself, the deterioration of the general condition and delays in the initiation of chemotherapy due to perforation contribute to the poor prognosis of intestinal T‐cell lymphoma.

As a limitation, genetic or molecular examinations have not been performed to diagnose MEITL. Further, we could not perform PET/CT and bone marrow puncture for the staging of MEITL due to the rapid deterioration of the patient's condition.

In conclusion, we present the case of MEITL causing jejunal perforation. MEITL is prone to cause intestinal perforation and the prognosis is very poor when it occurs. There is a necessity to establish a method to predict cases with intestinal perforation and systematize the treatment strategies to avoid perforation.

## Informed consent

Consent obtained directly from patient(s).
